# Morphology of the first three zoeal stages of the deep-sea caridean shrimp *Heterocarpus fascirostratus* Yang, Chan & Kumar, 2018 (Crustacea, Decapoda, Pandalidae)

**DOI:** 10.3897/zookeys.1042.64407

**Published:** 2021-06-04

**Authors:** Guo-Chen Jiang, Tin-Yam Chan

**Affiliations:** 1 Marine Ecology and Conservation Research Center, National Academy of Marine Research, Kaohsiung 80661, Taiwan National Academy of Marine Research Kaohsiung Taiwan; 2 Institute of Marine Biology and Center of Excellence for the Oceans, National Taiwan Ocean University, Keelung 202301, Taiwan National Taiwan Ocean University Keelung Taiwan

**Keywords:** Laboratory rearing, larval development, zoea

## Abstract

The larvae of the deep-sea pandalid shrimp *Heterocarpus fascirostratus* Yang, Chan & Kumar, 2018 were successfully hatched and cultured to the third zoeal stage. The larvae reached the third zoeal stage nine days after hatching at a water temperature of 21 ± 1 °C. Although members of *Heterocarpus* A. Milne-Edwards, 1881 have rather diverse body forms and are often separated into many species groups, the early zoeal morphology of *H. fascirostratus* follows the general developmental pattern of the species in *Heterocarpus.* The main differences amongst these larvae are body size, spines on the anteroventral margin of the carapace, and the endopod setation of the third maxilliped.

## Introduction

The deep-sea pandalid shrimp genus *Heterocarpus* A. Milne-Edwards, 1881 is often considered as having fishery potential (Holthuis 1980; Chan 1998). Thirty species are now known in the genus (Yang et al. 2018) and they have rather high morphological diversity with at least five species groups recognized to date according to the spination of the pleon and the lateral carinae on the carapace (Crosnier 1988; Yang et al. 2010, 2018; Liao et al. 2019). Larval development, however, has only been reported in four species of *Heterocarpus*; namely *H. abulbus* Yang, Chan & Chu, 2010 (Jiang et al. 2014 [Zoea I]; 2016 [ZI–ZIX]), *H. ensifer* A. Milne-Edwards, 1881 (Landeira et al. 2010 [ZI–ZIV]), *H. hayashii* Crosnier, 1988 (Jiang et al. 2014 [ZI]), and *H. sibogae* de Man, 1917 (Iwata et al. 1986 [ZI–ZV]; Jiang et al. 2014 [ZI]).

*Heterocarpus fascirostratus* Yang, Chan & Kumar, 2018 is the last species described in the genus and belongs to a different species group from those species of the same genus with known larval morphology. *Heterocarpus fascirostratus* only has pleonite III bearing an overhanging spine. *Heterocarpus ensifer*, *H. hayashii* and *H. sibogae* have posterior spines on both pleonites III and IV. *Heterocarpus abulbus* completely lacks a spine on the pleon. In the present work, an ovigerous female of *H. fascirostratus* was collected off the South China Sea and its hatching larvae were cultured for the first time until the third zoeal stage.

## Materials and methods

An ovigerous female of *H. fascirostratus* was captured by the research vessel ‘Ocean Researcher I’ (station CP 4128) with a French beam trawl at depths of 420–444 m off Dongsha Island (Pratas, Taiwan, 20°44.857'N, 116°08.010'E) in the South China Sea. The ovigerous female was maintained in sea water (35 ± 1‰ salinity) at 14 ± 1 °C in the laboratory. After hatching, ~ 200 actively swimming larvae each were transferred to two beakers (5 L) with aerated seawater, temperature at 21 ± 1 °C and natural photoperiod. The larvae were fed daily with *Artemia* nauplii and rotifers from Zoea I to III and with water changed every day. Specimens of each zoeal stage were collected immediately after the larvae molted and subsequently preserved in a 70% ethylene glycol solution. About five larvae were sampled each day to check for moults. At least two larvae from each stage were dissected, mounted on glass slides and examined under a stereo microscope (OLYMPUS SZX12) using fine entomological needles. Appendages were drawn using a *camera lucida* installed on a compound microscope (Olympus BX50). About 80% of the larvae developed to Zoea II, but only two larvae moulted to Zoea III and the rearing terminated then.

The descriptions and figures were arranged according to the standards proposed by Kamanli et al. (2018). Morphological terminology follows Yang and Ko (2004), Landeira et al. (2010), Clark and Cuesta (2015), Kamanli et al. (2018) and Almeida et al. (2021). Zoea I is completely described in detail; however, only appendage changes were described in the subsequent stages. Abbreviations of larval measurements are as follows: carapace length (**CL**), from the postorbital margin to the posteromedian end of the carapace; body length (**BL**), from the postorbital margin of the carapace to the posterior end of the telson; and total length (**TL**), from the tip of the rostrum to the tip of the telson. These are all given as mean values followed by the range (in parentheses). The parental female and larvae are deposited in the National Taiwan Ocean University (NTOU M02078).

## Results

### Larval descriptions

#### Zoea I (Fig. 1)

Period from hatching to end of instar: 1–5 days.

Size (*N* = 5): CL, 0.44 mm (0.41–0.47 mm); BL, 2.24 mm (2.16–2.35 mm); TL, 2.63 mm (2.51–2.76 mm).

**Figure 1.**
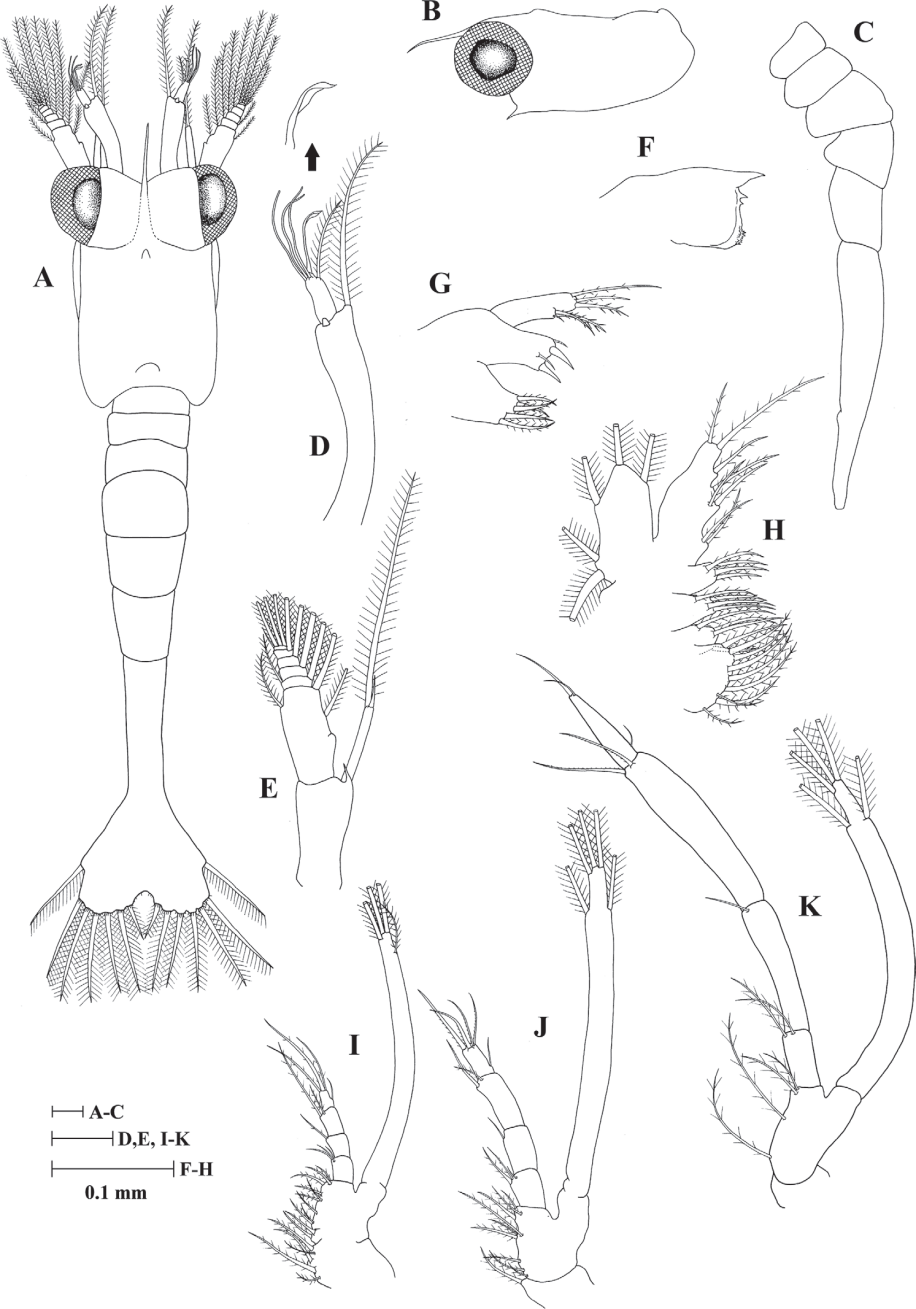
Zoea I of *Heterocarpus fascirostratus***A** dorsal view **B** carapace lateral view **C** pleon lateral view **D** antennule **E** antenna **F** mandible **G** maxillule **H** maxilla **I** first maxilliped **J** second maxilliped **K** third maxilliped.

Carapace (Fig. 1A, B): Slightly dorsoventrally flattened; rostrum slightly curved downwards, slender, shorter than antennular peduncle; dorsal anterior and posterior processes present; anteroventral margin only bearing strong pterygostomial spine; eyes sessile.

Antennule (Fig. 1D): Peduncle unsegmented, slender and terminally bearing one long plumose seta and a small tubercle; outer flagellum with one spatulate seta, three long aesthetascs and one plumose seta.

Antenna (Fig. 1E): Peduncle unsegmented with single basal spine near endopod; endopod unsegmented, with single long terminal plumose seta, one sharp, slender spine; exopod (scaphocerite) 6-segmented, bearing inner tubercle and with nine plumose setae on the inner margin (2,1,1,1,1,3), two plumose setae on the outer margin and one simple seta on apex (1,1,0,0,0,1).

Mandible (Fig. 1F): Palp absent; incisor and molar processes developed; *lacinia mobilis* present.

Maxillule (Fig. 1G): Coxal endite with five plumose setae; basial endite with two simple and two stout setae; endopod unsegmented with one small simple seta, one sparsely plumose seta subterminally, one sparsely hardy plumodenticulate seta and three terminal setae (one sparsely hardy plumodenticulate and two sparsely plumose); exopod absent.

Maxilla (Fig. 1H): Coxal endite bilobed with 9 + 4 setae (two papposerrate and eleven plumose); basial endite bilobed with 4 + 4 plumose setae; endopod 5-lobed, with nine (3 + 2 + 1 + 1 + 2) plumose setae; exopod (scaphognathite) margin with five plumose setae.

First maxilliped (Fig. 1I): Coxa with five setae (two papposerrate and three plumose); basis with eleven plumose setae; endopod 4-segmented, bearing 3 (simple), 1 (pappose), 2 (one papposerrate and one plumose), 4 (one median simple and three terminal sparsely plumose) setae; exopod unsegmented, armed distally with one subterminal and three plumose natatory setae.

Second maxilliped (Fig. 1J): Coxa with one plumose seta; basis with nine setae (one papposerrate and eight plumose); endopod 4-segmented, bearing 3 (one small simple, one pappose and one plumose), 1 (simple), 2 (one denticulate and one plumose), 5 (one subterminal simple and four terminal denticulate) setae; exopod unsegmented, armed distally with two subterminal and three plumose natatory setae.

Third maxilliped (Fig. 1K): Coxa without setae; basis with four plumose setae; endopod 4-segmented and slightly longer than exopod, with 2 (plumose), 1 (simple), 2 (denticulate), 3 (one subterminal simple and two terminal simple) setae; third segment slightly swollen compared to second segment; exopod unsegmented, armed distally with two subterminal and three plumose natatory setae.

Pereiopods: Absent.

Pleon (Fig. 1A, C): With five pleonites, sixth pleonite fused with telson; lacking setae or spines.

Pleopods: Absent.

Uropod: Absent.

Telson (Fig. 1A): Subtriangular, posterior margin with 7 + 7 plumose setae, outermost two pairs only plumose on inner margin; bases of each seta except outermost one with row of minute spinules.

#### Zoea II (Fig. 2)

Period from hatching to end of instar: 6–8 days.

Size (*N* = 2): CL, 0.50 mm (0.48–0.51 mm); BL, 2.52 mm (2.37–2.66 mm); TL, 2.74 mm (2.62–2.86 mm).

**Figure 2.**
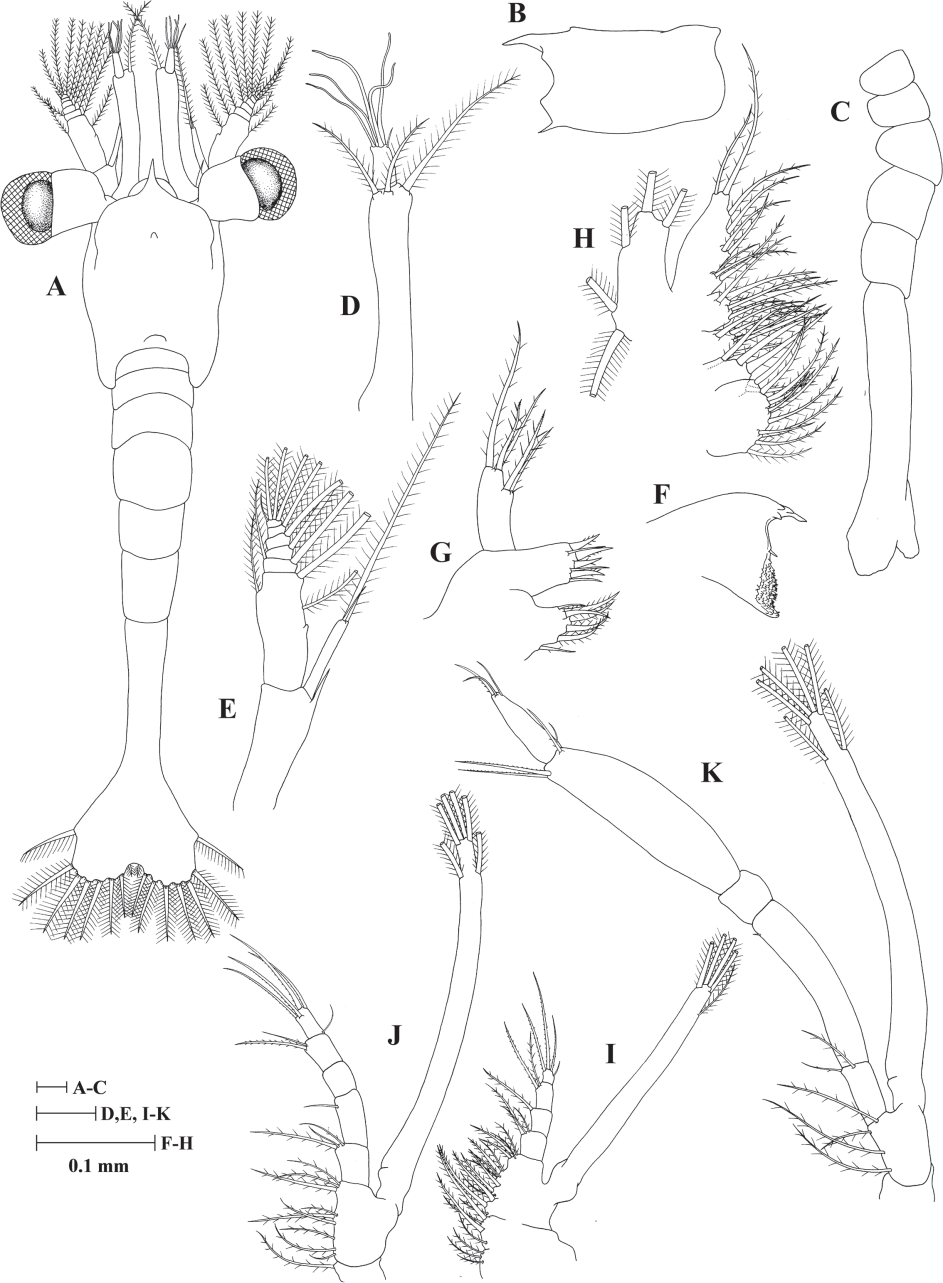
Zoea II of *Heterocarpus fascirostratus***A** dorsal view **B** carapace lateral view **C** pleon lateral view **D** antennule **E** antenna **F** mandible **G** maxillule **H** maxilla **I** first maxilliped **J** second maxilliped **K** third maxilliped.

Carapace (Fig. 2A, B): Rostrum shorter than in Zoea I and ~ 0.4 × as long as CL; eyes stalked, funnel-shaped.

Antennule (Fig. 2D): Peduncle unsegmented, bearing a pair of terminal plumose setae and one long plumose seta; outer flagellum with one simple seta and five long aesthetascs.

Mandible (Fig. 2F): Incisor and molar processes developed.

Maxillule (Fig. 2G): Coxal endite with seven setae (one simple, one pappose subterminal and five terminal plumose); basial endite with three simple setae and four cuspidate setae.

Maxilla (Fig. 2H): Coxal endite bilobed with 11 + 4 plumose setae; basial endite bilobed with 4 + 5 plumose setae.

First maxilliped (Fig. 2I): Coxa with seven plumose setae; basis with fourteen plumose setae; endopod 4-segmented, with 3 (one simple, one pappose and one plumose), 1 (plumose), 2 (one sparsely plumose and one plumose), 4 (one median simple and three terminal sparsely plumose) setae; exopod unsegmented, armed distally with one subterminal and four plumose natatory setae.

Second maxilliped (Fig. 2J): Endopod 5-segmented, with 3 (one simple, one pappose and one plumose), 1 (simple), 0, 2 (one denticulate and one plumose), 5 (one subterminal simple and four terminal denticulate) setae; exopod unsegmented, armed distally with two subterminal and four plumose natatory setae.

Third maxilliped (Fig. 2K): Endopod 5-segmented, with 2 (one pappose and one plumose), 1 (small simple), 0, 3 (one outer papposerrate and two inner denticulate), 4 (one subterminal simple and two terminal simple, one papposerrate) setae, third segment obvious swollen than second segment; exopod unsegmented, armed distally with two subterminal and four plumose natatory setae.

Telson (Fig. 2A): Posterior margin with 8 + 8 plumose setae.

#### Zoea III (Fig. 3)

Period from hatching: 9 days.

Size (*N* = 2): CL, 0.62 mm (0.60–0.63 mm); BL, 2.77 mm (2.71–2.82 mm); TL, 2.93 mm (2.86–2.99 mm).

**Figure 3.**
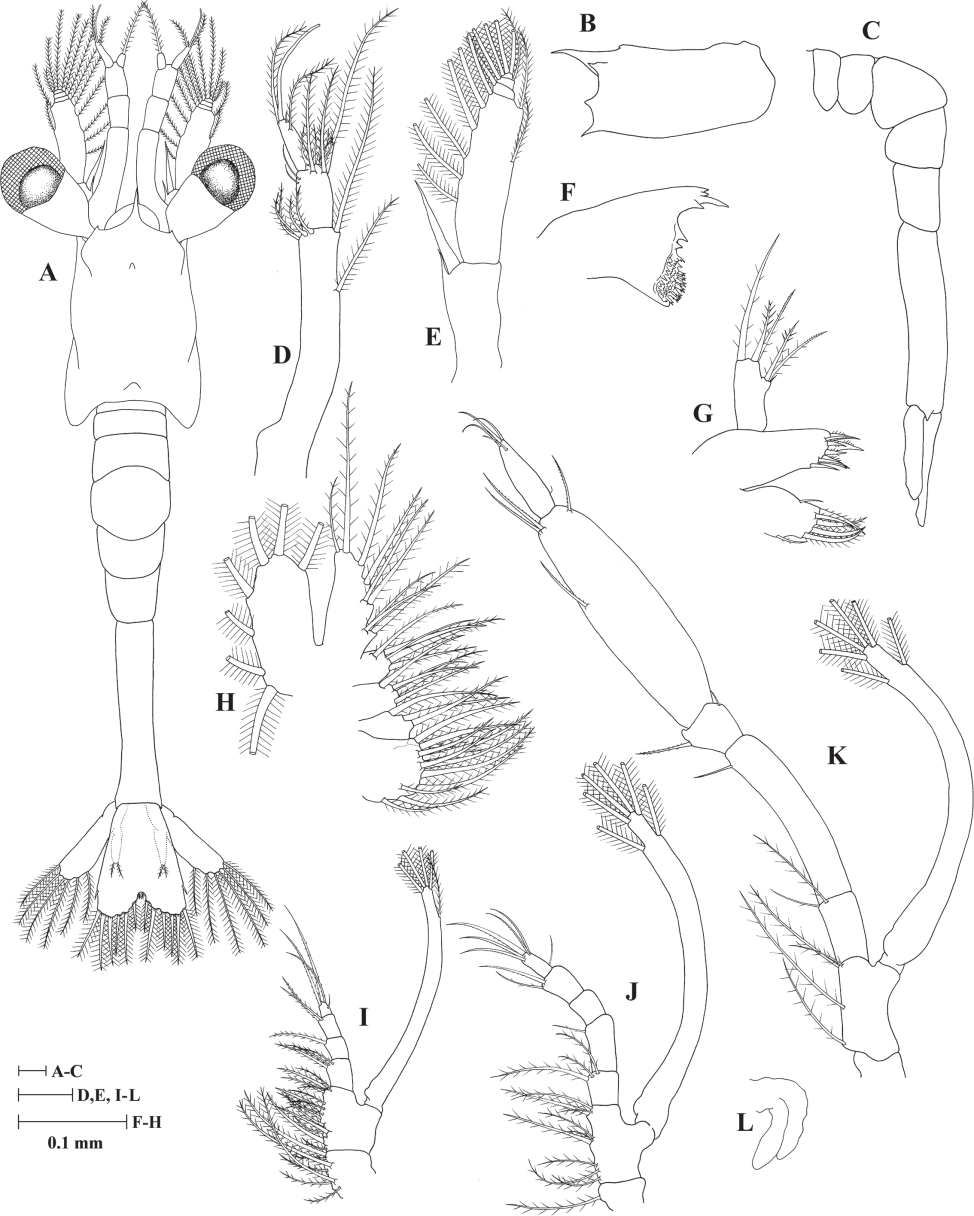
Zoea III of *Heterocarpus fascirostratus***A** dorsal view **B** carapace lateral view **C** pleon lateral view **D** antennule **E** antenna **F** mandible **G** maxillule **H** maxilla **I** first maxilliped **J** second maxilliped **K** third maxilliped **L** first pereiopod.

Carapace (Fig. 3A, B): Supraorbital spine present; rostrum short, ~ 0.35 × as long as CL.

Antennule (Fig. 3D): Peduncle 2-segmented, with the proximal segment bearing a small process and six plumose setae, arranged like 1+5; distal segment bearing nine setae (two simple and seven plumose); outer flagellum with one long terminal aesthetasc, two long plumose, one small simple seta.

Antenna (Fig. 3E): Endopod spine-like and without seta; exopod distally 4-segmented, with eleven plumose setae on inner margin (6,1,1,3), three plumose setae on outer margin.

Maxillule (Fig. 3G): Basial endite with three simple setae and five cuspidate setae.

Maxilla (Fig. 3H): Exopod margin with seven plumose setae.

Third maxilliped (Fig. 3K): Endopod 5-segmented with 2 (one pappose and one plumose), 1 (simple), 2 (one outer small simple and one inner denticulate), 4 (one outer and three inner denticulate), 5 (one outer small simple and four terminal simple) setae.

Pereiopods (Fig. 3L): First pereiopod as biramous bud.

Pleon (Fig. 3A, C): With six pleonites.

Uropod (Fig. 3A): Biramous; endopods rudimentary with two plumose setae; exopods well developed with six plumose setae.

Telson (Fig. 3A): Posterior margin with 8 + 8 setae, comprising seven pairs of sparsely plumose setae with outermost seta on each side shorter, simple, subterminal in position.

## Discussion

Previous studies together with the present work have shown the five species of *Heterocarpus* with known larval morphologies to belong to three different species groups: *H. abulbus* lacking spines on the pleon, *H. ensifer* / *H. hayashii* / *H. sibogae* bearing posterior spines on the pleonites III and IV, and *H. fascirostratus* only has pleonite III bearing an overhanging spine. Nevertheless, the general appearances of the early zoeal stages are rather similar amongst these five species. Thus, the early zoeal morphology of *H. fascirostratus* also has the common characters of pandalid larvae, such as the eye peduncle narrowed at the base, carapace with two dorsal protuberances and the anteroventral margin bearing spines, antennule with the peduncle strongly concave and exopod bearing spatulate seta, the antenna with a segmented exopod, and the rostrum elongated in earlier stages (see Thatje and Bacardit 2000; Landeira et al. 2010; Jiang et al. 2014). On the other hand, the first zoea of *H. fascirostratus* differs from the other four congeneric species in body size, spinulation of the carapace, and setation of third maxilliped (see Table 1). Moreover, the third maxilliped endopod has the third segment slightly swollen in *H. fascirostratus* but rather slender in the other four species.

**Table 1.** Characteristics of the first zoeae in five species of *Heterocarpus*.

Characters	*H. abulbus*	*H. ensifer*	*H. fascirostratus*	*H. hayashii*	*H. sibogae*
	(see Jiang et al. 2014, 2016)	(see Landeira et al. 2010)	(present study)	(see Jiang et al. 2014)	(see Jiang et al. 2014)
**Carapace length (mm)**	0.53–0.58	~ 0.42	0.41–0.47	0.38–0.45	0.38–0.43
**Anterolateral spines**	2	2	0	2	1
**Antennule**					
Peduncle tubercle	1 small tubercle	2 small tubercles	1 small tubercle	1 small tubercle	1 small tubercle
**Maxillule**					
Endopod setation	5	6	6	5	5
**Maxilla**					
Coxal endite setation	9+4	9+4	9+4	8+3	9+4
Basial endite setation	4+4	5+5	4+4	4+4	3+4
**First maxilliped**					
Coxal setation	5	7	5	4	4
Basial setation	11	12	11	10	9
**Second maxilliped**					
Coxal setation	2	1	1	1	1
Basial setation	8	9	9	9	6
Endopod setation	3,1,2,5	3,1,2,4	3,1,2,5	3,1,2,5	2,1,2,4
**Third maxilliped**					
Endopod setation	2,1,2,4	2,1,2,4	2,1,2,3	2,1,2,4	2,1,2,4

Although Iwata et al. (1986) reported on the ZI–ZV of *H. sibogae*, their description and illustrations are not detailed enough according to current standards for making comparisons (e.g., the presence of anteroventral spines on the carapace in ZI was not described; see Jiang et al. 2014). Therefore, the ZII and ZIII of *H. fascirostratus* can only be compared with those of *H. abulbus* and *H. ensifer*. They also differ in the number of spines on the anteroventral margin of carapace (none in *H. fascirostratus*, one in *H. abulbus*, two in *H. ensifer*), setation on the endopod of the third maxilliped (ZII and ZIII 2,1,0,3,4 and 2,1,2,4,5 setae, respectively for *H. fascirostratus*, vs. 2,1,0,2,4 and 2,1,1,2,5 respectively for the other two species), and the shape of the third maxilliped endopod (somewhat swollen in *H. fascirostratus*, vs. slender in the other two species). Moreover, in ZII the first segment of the endopod of the second maxilliped bears three setae in *H. fascirostratus* and *H. ensifer*, but four setae in *H. abulbus*.

Table 2 summarizes the characteristics of the three zoeal stages of *H. fascirostratus*. The major characters of each zoeal stage are:

**Table 2.** Characteristics of the first three zoeal stages of *Heterocarpus fascirostratus*. Abbreviations: a, aesthetascs; b, basal spine; c, cuspidate seta; p, plumose seta; pa, pappose seta; pe, papposerrate seta; s, spatulate seta; sh, sparsely hardy plumodenticulate; sp, sparsely plumose seta; lob, lobed; t, stout seta; seg, segment/segmented; v, slender spine; x, simple seta.

Appendage	Stages		
	Zoea I	Zoea II	Zoea III
**Carapace length (mm)**	0.41–0.47	0.48–0.51	0.60–0.63
**Antennule**			
Peduncle	1 small tubercle+1p	3p	6p, 2x+7p
Primary flagellum setation	3a+1p+1s	5a+1x	1a+2p+1x
**Antenna**			
Peduncle	1b	1b	1b
Endopod	1p+1v	1p+1v	0
Exopod	6-seg,11p+1x	6-seg, 11p+1x	4-seg, 14p
**Maxillule**			
Coxal endite setation	5p	7(1x+1pa+5p)	7(1x+1pa+5p)
Basial endite setation	2t+2x	4c+3x	5c+3x
Endopod setation	1x+1sp+1sh+3(1sh+2sp)	1x+1sp+1sh+3(1sh+2sp)	1x+1sp+1sh+3(1sh+2sp)
**Maxilla**			
Coxal endite setation	9p+4p (2pe+11p)	11p+4p	11p+4p
Basial endite setation	4p+4p	4p+5p	4p+5p
Endopod setation	5-lob, 3,2,1,1,2	5-lob, 3,2,1,1,2	5-lob, 3,2,1,1,2
Exopod setation	5p	5p	7p
**First maxilliped**			
Coxal setation	2pe+3p	7p	7p
Basial setation	11p	14p	14p
Endopod setation	4-seg, 3,1,2,4	4-seg, 3,1,2,4	4-seg, 3,1,2,4
Exopod setation	4p	5p	5p
**Second maxilliped**			
Coxal setation	1p	1p	1p
Basial setation	1pe+8p	1pe+8p	1pe+8p
Endopod setation	4-seg, 3,1,2,5	5-seg, 3,1,0,2,5	5-seg, 3,1,0,2,5
Exopod setation	5p	6p	6p
**Third maxilliped**			
Basial setation	4p	4p	4p
Endopod setation	4-seg, 2,1,2,3	5-seg, 2,1,0,3,4	5-seg, 2,1,2,4,5
Exopod setation	5p	6p	6p
**First pereiopod**	Absent	Absent	Biramous bud
**Uropod**			
Protopod	–	–	0
Endopod	–	–	2p
Exopod	–	–	6p
**Telson**	7p+7p	8p+8p	(1x+7p)+(1x+7p)

- ZI- eyes sessile, three pairs of maxillipeds, slender rostrum slightly curved, antennule peduncle unsegmented and bearing one small tubercle, pleon with five somites.
- ZII- eyes stalked, rostrum shorter than in ZI and ~ 0.4 × as long as CL, antennule peduncle bearing two plumose setae.
- ZIII- antennule peduncle two segmented, pleon with six somites, first pereiopod developed, uropods with exopods.
